# The effect of meditation on regulation of internal body states

**DOI:** 10.3389/fpsyg.2015.00924

**Published:** 2015-07-07

**Authors:** Sahib S. Khalsa, David Rudrauf, Richard J. Davidson, Daniel Tranel

**Affiliations:** ^1^Department of Neurology, University of IowaIowa City, IA, USA; ^2^Laureate Institute for Brain ResearchTulsa, OK, USA; ^3^Faculty of Community Medicine, University of TulsaTulsa, OK, USA; ^4^Laboratory of Functional Imaging, Institut National de la Santé et de la Recherche Médicale U678s/Université Pierre et Marie CurieParis, France; ^5^Department of Psychology, University of Wisconsin-MadisonMadison, WI, USA; ^6^Department of Psychology, University of IowaIowa City, IA, USA

**Keywords:** meditation, adrenergic, regulation, sympathetic, parasympathetic, isoproterenol, body

## Abstract

Meditation is commonly thought to induce physiologically quiescent states, as evidenced by decreased autonomic parameters during the meditation practice including reduced heart rate, respiratory rate, blood pressure, skin conductance, and increased alpha activity in the electroencephalogram. Preliminary empirical support for this idea was provided in a case report by Dimsdale and Mills (2002), where it was found that meditation seemed to regulate increased levels of cardiovascular arousal induced by bolus isoproterenol infusions. In that study, while meditating, a self-taught meditator exhibited unexpected decreases in heart rate while receiving moderate intravenous doses of the beta adrenergic agonist isoproterenol. This effect was no longer observed when the individual received isoproterenol infusions while not meditating. The current study was designed to explore this phenomenon empirically in a group of formally trained meditators. A total of 15 meditators and 15 non-meditators individually matched on age, sex, and body mass index were recruited. Participants received four series of infusions in a pseudorandomized order: isoproterenol while meditating (or during a relaxation condition for the non-meditators), isoproterenol while resting, saline while meditating (or during a relaxation condition for the non-meditators), and saline while resting. Heart rate was continuously measured throughout all infusions, and several measures of heart rate were derived from the instantaneous cardiac waveform. There was no evidence at the group or individual level suggesting that meditation reduced the cardiovascular response to isoproterenol, across all measures. These results suggest that meditation is not associated with increased regulation of elevated cardiac adrenergic tone.

## Introduction

Meditation is a form of mental training that has been practiced for thousands of years, and that can be conceptualized as a family of complex emotional and attentional regulatory training regimens developed for various ends, including the cultivation of well-being and emotional balance (Davidson et al., 1976; Ekman et al., 2005; Brefczynski-Lewis et al., 2007). Meditation has also been defined as involving a process of intentional self-regulation of attention, in which attention is directed from a combination of external and internal stimuli to a primarily internally perceptive state (Astin et al., 2003; Bonadonna, 2003). Traditional philosophies emphasize that anyone can learn to meditate (Taimni, 1961), and that through repeated practice meditation provides long-term effects that outlast the confines of individual meditative states (Nyanaponika, 1969; Ahir, 1999; Burley, 2000).

A central tenet of early investigations into the effects of meditation has been that meditation induces a physiologically quiescent bodily state. This was based on initial reports of decreases in autonomic parameters such as heart rate, respiratory rate, blood pressure, skin conductance, and adrenergic reactivity, as well as increased levels of alpha activity in the electroencephalogram during the practice of meditation [mostly Transcendental Meditation (TM)] (Wallace et al., 1971; Orme-Johnson, 1973; Beary and Benson, 1974; Farrow and Hebert, 1982; Hoffman et al., 1982; Jevning et al., 1992; Travis and Wallace, 1997; Aftanas and Golocheikine, 2002; Aftanas and Golosheykin, 2005). Early researchers described this pattern of autonomic responses as an integrated “relaxation response” (Benson et al., 1974). More recently, investigations have continued finding reductions in physiological parameters such as heart rate and blood pressure occur during the acute meditation practice (Telles et al., 1995; Barnes et al., 1999; Solberg et al., 2004) as well as following practice over longer periods of time (Barnes et al., 2004; Harinath et al., 2004). Similar effects have also been reported for more secular and abbreviated protocols such as an 8 week training in Mindfulness Based Stress Reduction, which incorporates instruction in meditation as well as teaches cognitive and other approaches to enhance stress reduction (Hughes et al., 2013).

Although the notion that meditation results in physiologically quiescent states is well established, the extent to which meditative practices exert such effects is unknown. Early investigations of yogis in India who claimed to be able to exert considerable voluntary control of the heart (some claimed to be able to stop the heart) revealed limited support for this idea. At best, some individuals were able to exert transient bradycardia, or reductions in heart rate by engaging in combinations of posture manipulation, muscular contraction and breath holding (including the Valsalva maneuver, or “bearing down,” which elicits a complex pattern of reflexes including tachy/bradycardia and hyper/hypotension) (Wenger et al., 1961). Subsequent studies have reported mixed results, primarily for different yoga adepts (Fenz and Plapp, 1970; Kothari et al., 1973). More recently, shorter term changes in the ability to reduce the resting heart rate have been reported (Telles et al., 2004). The perception that meditation and yoga confers an increased cardiac regulatory capability has persisted, despite this heterogeneous literature.

Preliminary empirical support via a case study approach suggested a novel effect of meditation on the ability to regulate increased levels of cardiovascular arousal. Dimsdale and Mills (2002) reported a case study in which a female meditator was randomly recruited to participate in a standardized isoproterenol challenge as a control subject in their studies of sympathetic nervous system regulation in hypertension (Dimsdale et al., 1988; Mills et al., 1998). Isoproterenol stimulates peripheral beta 1 and beta 2 adrenergic receptors equally, and when administered intravenously results in rapid and transient increases in heart rate, contractility and bronchodilation, as well as decreases in diastolic blood pressure. The protocol involved administration of a standard isoproterenol sensitivity test, involving sequentially increasing doses of intravenous isoproterenol. Halfway into the infusions the woman spontaneously started meditating, and continued to meditate during the infusions. At the time of the isoproterenol challenge the investigators did not know she was a meditator, but noticed afterwards that after the fourth dose (1 mcg) her heart rate had begun to decrease, in stark contrast to the expected increase. They report that at the highest dose (4 mcg), when the expected heart rate response was a 21 beats per minute (bpm) increase above baseline, they observed a 17 bpm *decrease* below baseline. When this unexpected result was mentioned, the participant stated that she had decided to start meditating halfway into the experiment. Suspecting that her meditation practice had interfered with the isoproterenol response, she was asked to return 2 weeks later and repeat the challenge, with explicit instructions to avoid meditating during the infusions. Her heart rate response to the subsequent infusion protocol appeared entirely consistent with the typical laboratory response to isoproterenol at rest (i.e., an approximately 20 bpm increase above baseline at the highest dose). This individual reported a self-taught meditation practice for many years, and that she did not practice any particular tradition of meditation.

The current study was designed to investigate whether there is an effect of meditation on attenuating adrenergically mediated increases in sympathetic arousal, using an empirical group study approach. We recruited formally trained meditators with at least several years of experience, and examined autonomic parameters of sympathetic arousal during meditation and resting conditions, during the application of isoproterenol and saline infusions. To examine whether such a putative effect was specific to a meditation practice or was a generic ability inherent to humans more generally, we also recruited a group of individually matched non-meditators, and tested them under relaxation and resting conditions.

We hypothesized that the practice of meditation would be specifically associated with an enhanced ability to regulate body states occurring in the context of acute physiological arousal. To test this hypothesis, the impact of a meditation practice on the cardiovascular response to isoproterenol was assessed. Several specific predictions derive from our hypothesis: (1) In the face of isoproterenol infusions, during a meditation practice meditators would display lower isoproterenol induced heart rate increases than during a non-meditation condition. (2) Meditators would demonstrate lower heart rate increases during a meditation practice than non-meditators during a relaxation practice. (3) Heart rate reductions in the meditators would be specific to meditation, i.e., meditators and non-meditators would have equivalent heart rate responses to isoproterenol at rest (when not engaging in meditation or relaxation strategies).

## Methods

### Participants

Fifteen meditators and fifteen non-meditators participated in the study. Each non-meditator was individually matched to a corresponding meditator based on three criteria: age, gender, and body mass index (see Table 1). Meditators were considered eligible for participation if they reported a continuous (daily or near daily) meditation practice during the previous 2 years, and if they had also attended one or more weeklong meditation retreats within the previous year. Using this criteria, 11 of the recruited meditators were Vipassana practitioners, and the other four meditators were Kundalini practitioners. Non-meditators were considered eligible for participation if they had never received formal meditation training in meditation or yoga and did not practice self-taught meditation.

**Table 1:** **Meditator and non-meditator demographic data**.

	**Meditators (M)**	**Non-meditators (NM)**
Sex	10 Men, 5 Women	10 Men, 5 Women
Age (yrs)	44.7 ± 13.2	44.0 ± 13.7
Body Mass Index	24.5 ± 4.6	25.5 ± 4.0
Race	15 Caucasian American	14 Caucasian American, 1 Asian American
Education (years)	17.3 ± 2.2	15.9 ± 2.3
Beck Anxiety Inventory score	5.1 ± 3.4	3.5 ± 2.9
Beck Depression Inventory score	4.3 ± 4.6	3.7 ± 5.3
Meditation practice (years)^*^	10.8 ± 10.8	0 ± 0
Cumulative meditation practice (hours)^*^	4947 ± 6251	0 ± 0
Retreat experience (days)^**^	19 ± 14	0 ± 0
CD25 (micrograms)	4.48 ± 1.5	4.72 ± 2.2
Practice similarity: iso + meditation/relaxation	3.5 ± 1.1	3.4 ± 1.1
Practice similarity: saline + meditation/relaxation	3.7 ± 1.1	3.8 ± 0.8

*Chronotropic Dose 25 (CD25): isoproterenol dose (mcg) required to increase the heart rate by 25 beats per minute. Mean ± SD*.

* *p < 0.01*,

** *p < 0.05*.

*Means ± standard deviation. Iso, isoproterenol*.

All participants were screened for the presence of any neurological, psychiatric, cardiac or respiratory disease during a detailed phone interview, and were excluded if they reported a history of disease in any of these categories. None of the study participants were smokers, and none of the women took oral contraceptives or were pregnant, as assessed via urine pregnancy test. Each participant demonstrated a normal 12 lead electrocardiogram (EKG), as assessed by a board certified cardiologist or neurologist.

This study was approved by the University of Iowa's Institutional Review Board, and all participants provided informed consent prior to participation.

### Tasks

Participants were informed that they would be asked to rest quietly with their eyes open on two occasions, and to meditate (or alternatively, to relax) with their eyes closed on two occasions. The instruction to keep the eyes open or closed was chosen to reflect the manipulation reported in Dimsdale and Mills (2002). It was also chosen to improve the face validity of the meditation and relaxation conditions, as these activities are typically taught and practiced with the eyes closed. Meditators were asked to engage in their usual daily meditation practice for a period of 15–20 min during the meditation condition. Since the Kundalini tradition offers many different types of meditations deriving different types of effects, Kundalini meditators were asked to select a specific meditation practice that they had found helped them decrease their level of bodily arousal in the past. Since the Vipassana tradition does not provide such an approach, Vipassana meditators were simply asked to engage in their usual meditation practice. Non-meditators were instructed in the performance of a cognitive relaxation strategy for a period of 15–20 min during the relaxation condition. Specifically, they were asked to engage in a relaxation practice they had found useful for themselves in the past, one that would help them to “relax your mind and body, help you slow your thoughts, slow your breathing, and slow your heart rate.” If a non-meditator reported no such a strategy, they were given the option to perform one or any combination of several relaxation strategies including (1) reliving a pleasant memory, such as a warm day at the beach, (2) replaying their favorite song internally, (3) completely relaxing their musculature and/or (4) slowing their breathing. These strategies were selected with the aim of facilitating elicitation of the relaxation response (Benson et al., 1974). Non-meditators were specifically instructed to avoid falling asleep during the relaxation practice, despite any inclinations that might arise.

During the rest conditions both groups were instructed to avoid engaging in their meditation/relaxation practice, and instead, to explore their everyday thoughts (for example, thoughts about activities from the recent past such as what they had done the day before, or thoughts about potential future activities such as what they would do once the study had been concluded).

All participants were informed that they would be receiving isoproterenol or saline at some points during the entire session, but that neither they nor the experimenter would know when the nurse administered a particular agent. The room in which the study took place was kept quiet and was dimly lit throughout duration of the study to minimize distractions. Participants were seated in a padded chair, with a curtain draped over the arm containing the intravenous line, in order to reduce distraction and to preserve the blinding.

To assess task validity, after each meditation condition was complete, participants were asked to “rate how similar your meditation practice just now compares to your meditation practice in general,” on a scale from 1 (“not at all the same”) to 5 (“completely the same”). Non-meditators were also asked to rate their relaxation practice in a similar fashion, using the same scale. If they reported never having practiced the specific instructed relaxation condition, they were asked to relate their experience to other events where they had felt a state of relaxation.

### Infusion protocol

Two sets of standard bolus isoproterenol infusion protocols and two sets of matched saline infusion protocols were administered. The isoproterenol protocols consisted of sequentially increasing bolus isoproterenol doses of 0.1, 0.5, 1.0, 2.0, and 4.0 (mcg), delivered 3.5 min apart. The saline protocols consisted of five identically delivered bolus infusions of saline.

### Infusion delivery

Each infusion (isoproterenol and saline) consisted of two 3 milliliter (ml) bolus infusions delivered sequentially through an intravenous catheter. During isoproterenol infusions, a 3 ml bolus containing the specified dose was delivered, immediately followed by a 3 ml bolus of saline to flush the line. During saline infusions, a 3 ml bolus of saline was delivered, immediately followed by an additional 3 ml bolus of saline. Both bolus volumes were administered in entirety within a 15 s period by a nurse from the General Clinical Research Center. This method of delivery minimized the participant's ability to use external cues to distinguish between the different infusion types, and ensured rapid and standardized systemic introduction of isoproterenol.

### Procedure

The infusion protocol order was pseudorandomized and single blinded. This was necessitated by practical considerations: in order to ensure the safety of isoproterenol administration, a physician was required to be present during the first round of infusions. Due to constraints in the physician's schedule, a majority of the time the first condition consisted of isoproterenol infusions. However, the remaining task selection process (for example, isoproterenol plus rest vs. isoproterenol plus meditation) remained randomized, and was determined by the nurse administering the infusions. The remaining three infusion conditions were completely randomized.

### Psychophysiological measures

Heart rate was continuously measured throughout all infusions, from a lead II electrocardiogram (MP100 acquisition unit, Biopac Systems, Inc.), at a sampling rate of 200 Hz. All artifacts affecting the cardiac waveform (e.g., movement related and cardiac, such as premature ventricular contractions) were visually identified and manually removed.

**Figure 1:**
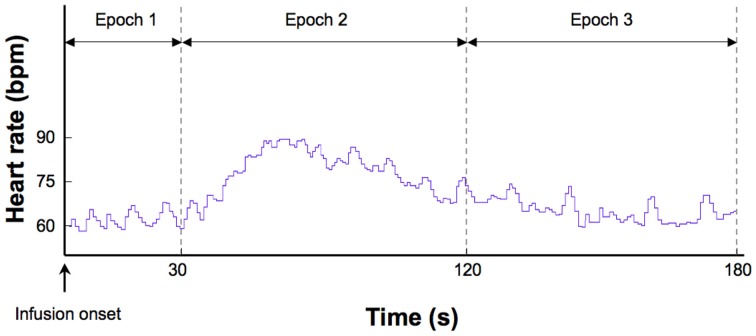
**Epochs used to derive different measures of heart rate**. This example shows a typical heart rate response to a 2.0 mcg dose of isoproterenol in a single subject.

Several measures of heart rate were derived from the instantaneous cardiac waveform. These measures were divided across three epochs of specific relevance to the timeline of heart rate changes induced by isoproterenol (Figure 1). The first epoch consisted of a 30 s interval starting immediately after the onset of each infusion. This reflects a period when bolus isoproterenol induced heart rate changes are unlikely to occur. The second epoch consisted of a 90 s interval beginning 30 s after infusion administration. This reflects a period when bolus isoproterenol induced heart rate changes are most likely to occur. The third epoch consisted of a 60 s period beginning immediately after the end of the second epoch. This interval reflects a period when the heart rate is nearing baseline or has already returned back to baseline. These three epochs combine to represent the 3 min following each infusion onset, when the probability of isoproterenol induced changes in heart rate are maximal. Because the participant could hear the nurse was preparing the next infusion during the final 30 s period (of the 3.5 min separating each infusion), this period was not included in the analysis to remove any potential influence of this preparatory period on the heart rate.

Within each epoch, four measures were obtained. (1) Mean heart rate change. This was determined for each participant by subtracting the average heart rate during epoch 1 from epoch 2. This controls for any potential residual elevations in baseline heart rate that might occur following increasing doses, and provided a reliable estimate of changes induced by isoproterenol administration. (2) Mean heart rates for each participant were determined across epochs 1, 2, and 3. This provided a measure of average heart rate over the entire window of response to isoproterenol. (3) The lowest heart rates occurring for each participant within a 3 s period throughout epochs 1, 2, and 3. This provided a measure of the floor effect for the heart rate response to isoproterenol. (4) The highest heart rates occurring for each participant within a 3-s period were identified across epochs 1, 2, and 3. This provided a measure of the ceiling effect of the heart rate response to isoproterenol.

Finally, heart rate and blood pressure were measured with an automated non-invasive blood pressure monitoring device (inflatable cuff wrapped around the dominant arm) similar to what was likely used during the Dimsdale and Mills (2002) study^1^. These latter measures were initiated 30 s after the start of each infusion, in an effort to mirror the type of measurement used by Dimsdale and Mills (2002). Due to variability in the amount of time taken for the machine to generate a blood pressure reading, each measure with this device was obtained approximately 60–80 s after each infusion onset (maximum range observed: 58–93 s after initiating measurement).

### Data analysis

Continuous data were analyzed using general linear models (GLM) with repeated measures, with group (meditator, non-meditator) as the between subjects factor and with condition (isoproterenol plus meditation or relaxation, isoproterenol plus rest, saline plus meditation or relaxation, saline plus rest) and dose (isoproterenol, saline) as the within subjects factors. In the GLM analysis, the meditation vs. rest contrast is explicitly evaluated in the condition term. Therefore, the pertinent measures determining whether meditators show differences in their autonomic responses during meditation vs. rest were tested by the group x condition, and by the group x condition x dose interactions. All repeated measures tests were assessed for violations of the sphericity assumption, and when violated, were corrected with the Huynh-Feldt method. In these instances the corrected *p*-values are reported, along with the Huynh-Feldt epsilon (ε) correction.

## Results

### Participants

Meditators reported significantly more years of meditation practice *t*_(28)_ = 3.90, *p* = 0.002, hours of cumulative meditation practice *t*_(28)_ = 3.07, *p* = 0.008, and days of retreat experience *t*_(28)_ = 2.31, *p* = 0.037, than non-meditators. The groups did not differ with respect to age *t*_(28)_ = 0.15, *p* = 0.88, body mass index *t*_(28)_ = −0.63, *p* = 0.53, or education *t*_(28)_ = 1.64, *p* = 0.11. The groups also did not differ with respect to baseline levels of anxiety (assessed via the Beck Anxiety Inventory) *t*_(28)_ = 1.39, *p* = 0.18, or depression (assessed via the Beck Depression Inventory) *t*_(28)_ = 0.33, *p* = 0.74.

### Mean heart rate change

**Figure 2:**
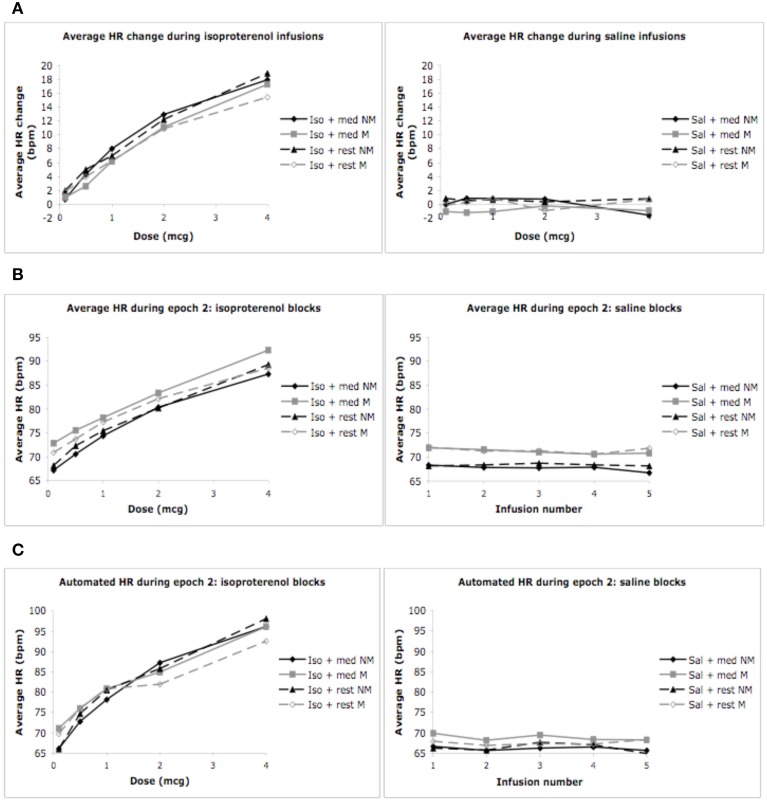
**Mean heart rates for both groups during mediation/relaxation and rest. (A)** Mean heart rate change (epoch 2 minus epoch 1). **(B)** Mean heart rate during epoch 2. **(C)** Mean heart rates measured via automated blood pressure monitor. For purposes of clarity, mean values are displayed without error bars, and the relaxation condition for the non-meditators is labeled as meditation.

As expected, we observed a significant effect of condition *F*_(1, 3)_ = 140.22, *p* < 0.0001, η^2^_*p*_ = 0.83, ε = 0.780, observed power = 1.00, and dose *F*_(1, 4)_ = 68.5, *p* < 0.0001, η^2^_*p*_ = 0.71, ε = 0.554, observed power = 1.00, on the mean heart rate response to isoproterenol. There was a significant interaction between condition and dose *F*_(1, 12)_ = 68.54, *p* < 0.0001, η^2^_*p*_ = 0.59, ε = 0.737, observed power = 1.00, such that increases in heart rate occurred at increasing doses of isoproterenol (but not saline) administration. However, despite these changes, there were no group differences in the heart rate response to isoproterenol. There was no effect of group *F*_(1, 28)_ = 2.97, *p* = 0.10, η^2^_*p*_ = 0.10, observed power = 0.384, and there were no interactions between condition and group *F*_(1, 3)_ = 0.21, *p* = 0.84, η^2^_*p*_ = 0.01, observed power = 0.088, between dose and group *F*_(1, 4)_ = 0.21, *p* = 0.84, η^2^_*p*_ = 0.01, observed power = 0.093, or between condition and group and dose *F*_(1, 12)_ = 0.82, *p* = 0.60, η^2^_*p*_ = 0.03, observed power = 0.482, suggesting that the heart rate increases induced by isoproterenol were not statistically different between the groups (Figure 2A). Mean average heart rate changes and associated 95% confidence intervals are displayed in Table 2.

**Table 2:** **Average heart rate changes and 95% confidence intervals across all conditions**.

**Average HR change**								
**Dose (mcg)**	**Non-meditators**		**95% CI**		**Meditators**		**95% CI**	
	**Mean**	**SE**	**Lower**	**Upper**	**Mean**	**SE**	**Lower**	**Upper**
**ISO + RELAXATION**					**ISO + MEDITATION**			
0.1	0.74	0.84	−0.99	2.47	0.97	0.84	−0.76	2.7
0.5	4.33	0.77	2.74	5.91	2.6	0.77	1.03	4.2
1	8.02	1.03	5.91	10.13	6.2	1.03	4.09	8.3
2	12.91	1.63	9.58	16.25	11.11	1.63	7.79	14.45
4	17.93	2.26	13.3	22.56	17.27	2.26	12.64	21.89
**ISO + REST**					**ISO + REST**			
0.1	1.97	0.65	0.65	3.31	1.82	0.65	0.48	3.15
0.5	5.01	0.85	3.28	6.74	3.97	0.85	2.24	5.7
1	6.99	0.76	5.44	8.53	6.28	0.76	4.73	7.82
2	12.22	1.39	9.38	15.06	10.89	1.39	8.05	13.73
4	18.85	1.54	15.71	22.02	15.44	1.54	12.28	18.59
**SALINE + RELAXATION**					**SALINE + MEDITATION**			
0.1	0	0.96	−1.96	1.97	−1.04	0.96	−3	0.92
0.5	0.89	0.66	−0.47	2.2	−1.17	0.66	−2.52	0.18
1	0.86	0.51	−0.19	1.91	−1.03	0.51	−2.08	0.02
2	0.76	0.52	−0.31	1.84	−0.22	0.52	−1.29	0.85
4	−1.54	1.04	−3.67	0.6	−0.88	1.04	−3.02	1.25
**SALINE + REST**					**SALINE + REST**			
0.1	0.85	0.59	−0.37	2.06	−0.13	0.59	−1.35	1.08
0.5	0.58	0.52	−0.49	1.65	0.33	0.52	−0.74	1.4
1	0.69	0.65	−0.62	2	0.88	0.64	−0.42	2.19
2	0.39	0.9	−1.47	2.24	−0.9	0.9	−2.75	0.95
4	0.82	0.61	−0.44	2.07	0.74	0.61	−0.51	2

### Mean heart rate

#### Epoch 1

We did not find an effect of condition on the average heart rate during epoch 1, *F*_(1, 3)_ = 0.39, *p* = 0.76, η ^2^_*p*_ = 0.01, observed power = 0.12, but did observe a significant effect of dose *F*_(1, 4)_ = 4.74, *p* = 0.008, η^2^_*p*_ = 0.15, ε = 0.616, observed power = 0.80. There was also a significant interaction between condition and dose *F*_(1, 12)_ = 4.41, *p* < 0.0001, η^2^_*p*_ = 0.14, ε = 0.772, observed power = 0.99, suggesting that increases in mean heart rate during epoch 1 occurred at increasing doses of isoproterenol (but not saline) administration. However, despite these changes, there were no group differences in heart rate during this period. There was no effect of group *F*_(1, 28)_ = 1.20, *p* = 0.28, η^2^_*p*_ = 0.04, observed power = 0.19, and there were no interactions between condition and group *F*_(1, 3)_ = 2.00, *p* = 0.12, η^2^_*p*_ = 0.07, observed power = 0.50, between dose and group *F*_(1, 4)_ = 0.33, *p* = 0.76, η^2^_*p*_ = 0.01, observed power = 0.12, or between condition and group and dose *F*_(1, 12)_ = 0.74, *p* = 0.68, η^2^_*p*_ = 0.03, observed power = 0.43, suggesting that the mean heart rate increases observed for increasing doses of isoproterenol during epoch 1 were not statistically different between groups. Average heart rate changes and associated 95% confidence intervals are displayed in Table 3. The finding of dose related increases during this epoch reflects that fact that some of the heart rate changes induced by isoproterenol (particularly at the higher doses) began occurring early, as was observed in several participants.

**Table 3:** **Average heart rates during epoch 1, including 95% confidence intervals**.

**Average HR—epoch 1**								
**Dose (mcg)**	**Non-meditators**		**95% CI**		**Meditators**		**95% CI**	
	**Mean**	**SE**	**Lower**	**Upper**	**Mean**	**SE**	**Lower**	**Upper**
**ISO + RELAXATION**					**ISO + MEDITATION**			
0.1	66.47	2.54	61.27	71.77	71.86	2.54	66.67	77.06
0.5	66.26	2.83	60.46	72.07	72.92	2.83	67.11	78.72
1	66.37	2.68	60.89	71.86	71.97	2.68	66.49	77.46
2	67.48	2.76	61.83	73.12	72.22	2.76	66.57	77.87
4	69.4	3.12	63	75.8	75.05	3.12	68.65	81.45
**ISO + REST**					**ISO + REST**			
0.1	66.18	2.52	61.03	71.35	69.09	2.52	63.92	74.25
0.5	67.32	2.59	62.02	72.2	69.74	2.59	64.44	75.04
1	68.46	2.6	64.12	73.79	70.98	2.6	65.65	76.32
2	67.98	2.65	62.58	73.29	71.28	2.64	65.87	76.69
4	70.45	2.97	64.37	76.53	73.11	2.97	67.03	79.19
**SALINE + RELAXATION**					**SALINE + MEDITATION**			
0.1	68.29	3.3	61.54	75.04	72.96	3.3	66.2	79.71
0.5	66.91	2.72	61.33	72.49	72.72	2.72	67.14	78.3
1	66.87	2.71	61.32	72.43	72.01	2.71	66.46	77.57
2	67.07	2.77	61.39	72.74	70.81	2.77	65.13	76.49
4	68.24	3.1	61.88	74.59	71.7	3.1	65.34	78.06
**SALINE + REST**					**SALINE + REST**			
0.1	67.26	2.78	61.56	72.96	72.16	2.78	66.46	77.86
0.5	67.73	2.67	62.27	73.19	70.91	2.67	65.45	76.37
1	68.00	2.73	62.42	73.59	70.42	2.73	64.83	76
2	67.94	2.87	62.05	73.83	71.43	2.87	65.54	77.32
4	67.31	2.58	62.02	72.59	71.1	2.58	65.81	76.38

#### Epoch 2

We observed a significant effect of condition on the average heart rate during epoch 2, *F*_(1, 3)_ = 84.19, *p* < 0.0001, η^2^_*p*_ = 0.75, observed power = 1.00, as well as for dose *F*_(1, 4)_ = 84.19, *p* < 0.0001, η^2^_*p*_ = 0.75, ε = 0.402, observed power = 1.00. There was also a significant interaction between condition and dose *F*_(1, 12)_ = 72.91, *p* < 0.0001, η ^2^_*p*_ = 0.72, ε = 0.330, observed power = 1.00, suggesting that increases in mean heart rate during epoch 2 occurred at increasing doses of isoproterenol (but not saline) administration. However, despite these changes, there were again no group differences in heart rate during this period. There was no effect of group *F*_(1, 28)_ = 0.65, *p* = 0.43, η^2^_*p*_ = 0.02, observed power = 0.12, and there were no interactions between condition and group *F*_(1, 3)_ = 2.00, *p* = 0.12, η^2^_*p*_ = 0.07, observed power = 0.37, between dose and group *F*_(1, 4)_ = 1.44, *p* = 0.24, η^2^_*p*_ = 0.05, observed power = 0.16, or between condition and group and dose *F*_(1, 12)_ = 0.70, *p* = 0.59, η^2^_*p*_ = 0.02, observed power = 0.41, suggesting that the mean heart rate increases observed for increasing doses of isoproterenol during epoch 2 were not statistically different between groups (Figure 2B and Table 4).

**Table 4:** **Average heart rates during epoch 2, including 95% confidence intervals**.

**Average HR—epoch 2**								
**Dose (mcg)**	**Non-meditators**		**95% CI**		**Meditators**		**95% CI**	
	**Mean**	**SE**	**Lower**	**Upper**	**Mean**	**SE**	**Lower**	**Upper**
**ISO + RELAXATION**					**ISO + MEDITATION**			
0.1	67.21	2.55	61.98	72.43	72.84	2.55	67.61	78.06
0.5	70.59	2.71	65.04	76.13	75.53	2.71	69.98	81.08
1	74.39	2.95	68.35	80.43	78.17	2.95	72.13	84.21
2	80.39	3.5	73.22	87.55	83.34	3.5	76.17	90.5
4	87.33	4.14	78.87	95.8	92.32	4.14	83.85	100.79
**ISO + REST**					**ISO + REST**			
0.1	68.17	2.51	63.03	73.31	70.9	2.51	65.76	76.04
0.5	72.34	2.64	66.93	77.74	73.71	2.64	68.31	79.12
1	75.44	2.84	69.62	81.26	77.26	2.84	71.44	83.08
2	80.21	3.19	73.66	86.75	82.17	3.19	75.63	88.71
4	89.31	3.51	82.13	96.49	88.55	3.51	81.37	95.73
**SALINE + RELAXATION**					**SALINE + MEDITATION**			
0.1	68.39	2.9	62.36	74.23	71.92	2.9	65.98	77.85
0.5	67.79	2.69	62.23	73.3	71.55	2.69	66.04	77.06
1	67.73	2.8	62.01	73.45	70.99	2.8	65.26	76.71
2	67.83	2.75	62.2	73.46	70.59	2.75	65.96	76.22
4	66.7	2.73	61.11	72.29	70.82	2.73	65.23	76.4
**SALINE + REST**					**SALINE + REST**			
0.1	68.10	2.77	62.44	73.77	72.03	2.77	66.36	77.7
0.5	68.31	2.63	62.93	73.7	71.24	2.63	65.86	76.63
1	68.69	2.69	63.19	74.19	71.3	2.66	65.8	76.8
2	68.33	2.66	62.89	73.77	70.53	2.66	65.09	75.97
4	68.12	2.8	62.38	73.86	71.84	2.8	66.1	77.58

#### Epoch 3

We observed a significant effect of condition on the average heart rate during epoch 3, *F*_(1, 3)_ = 8.30, *p* < 0.0001, η^2^_*p*_ = 0.23, observed power = 0.98, as well as for dose *F*_(1, 4)_ = 30.66, *p* < 0.0001, η^2^_*p*_ = 0.52, ε = 0.440, observed power = 1.00. There was also a significant interaction between condition and dose *F*_(1, 12)_ = 20.72, *p* < 0.0001, η^2^_*p*_ = 0.43, ε = 0.418, observed power = 1.00, suggesting that increases in mean heart rate during epoch 3 occurred at increasing doses of isoproterenol (but not saline) administration. However, despite these changes, there were no group differences in heart rate during this period. There was no effect of group *F*_(1,28)_ = 0.92, *p* = 0.35, η ^2^_*p*_ = 0.03, observed power = 0.15, and there were no interactions between condition and group *F*_(1, 3)_ = 1.68, *p* = 0.18, η ^2^_*p*_ = 0.06, observed power = 0.43, between dose and group *F*_(1, 4)_ = 0.93, *p* = 0.39, η ^2^_*p*_ = 0.03, observed power = 0.29, or between condition and group and dose *F*_(1, 12)_ = 0.65, *p* = 0.67, η^2^_*p*_ = 0.02, observed power = 0.38, suggesting that the mean heart rate increases observed for increasing doses of isoproterenol during epoch 3 were not statistically different between groups. The finding of dose related increases during this epoch reflects that fact that some of the heart rate changes induced by isoproterenol (particularly at the higher doses) were still present and in the process of returning to baseline, as was observed in several participants (Table 5).

**Table 5:** **Average heart rates during epoch 3, including 95% confidence intervals**.

**Average HR—epoch 3**								
**Dose (mcg)**	**Non-meditators**		**95% CI**		**Meditators**		**95% CI**	
	**Mean**	**SE**	**Lower**	**Upper**	**Mean**	**SE**	**Lower**	**Upper**
**ISO + RELAXATION**					**ISO + MEDITATION**			
0.1	66.58	2.58	61.29	71.87	71.57	2.58	66.28	76.85
0.5	66.28	2.71	60.73	71.82	71.7	2.71	66.16	77.25
1	68.1	2.74	62.49	73.71	72.5	2.74	66.89	78.11
2	70.59	3.15	64.13	77.05	75.11	3.15	68.66	81.57
4	73.94	3.67	66.43	81.45	81.28	3.67	73.77	88.79
**ISO + REST**					**ISO + REST**			
0.1	67.88	2.55	62.66	73.1	71.8	2.55	66.57	77.02
0.5	68.38	2.63	62.99	73.77	70.67	2.63	65.28	76.06
1	68.75	2.65	63.32	74.19	70.86	2.65	65.43	76.3
2	71.47	3.04	65.25	77.69	75.57	3.04	69.35	81.79
4	77.07	3.33	70.24	83.9	80.35	3.33	73.52	87.18
**SALINE + RELAXATION**					**SALINE + MEDITATION**			
0.1	67.93	2.74	62.31	73.54	71.97	2.74	66.34	77.57
0.5	68.06	2.67	62.6	73.52	71.85	2.67	66.39	77.31
1	68.58	2.7	63.04	74.12	71.2	2.7	65.66	76.74
2	67.89	2.78	62.2	73.58	70.39	2.78	64.7	76.08
4	66.48	2.69	60.97	72	70.09	2.69	65.57	75.6
**SALINE + REST**					**SALINE + REST**			
0.1	68.12	2.6	62.8	73.44	71.78	2.6	66.46	77.1
0.5	68.67	2.67	63.19	74.14	71.24	2.67	65.77	76.72
1	69.73	2.53	64.54	74.93	71.04	2.53	65.85	76.23
2	68.38	2.66	62.94	73.83	71.83	2.66	66.39	77.27
4	69.81	2.77	64.14	75.47	72.15	2.77	66.49	77.82

### Highest and lowest heart rates

**Figure 3:**
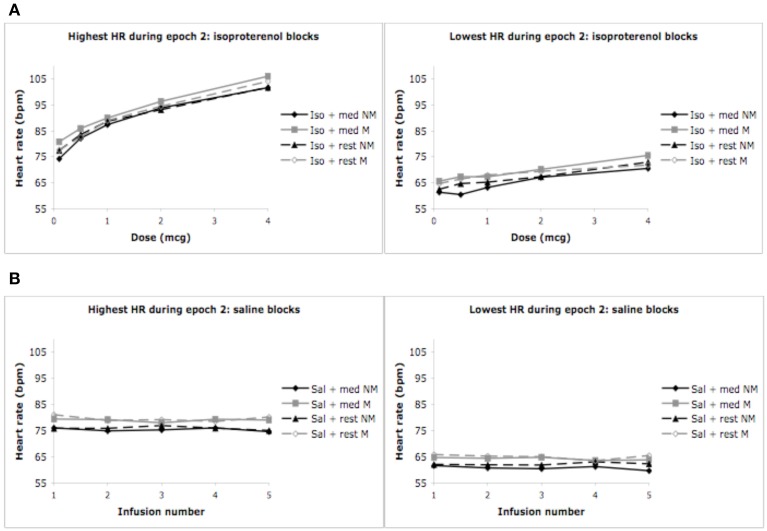
**Highest and lowest heart rates for both groups during epoch 2. (A)** Highest heart rates observed during a 3 s period, averaged across each group (ceiling effect). **(B)** Lowest heart rates observed during a 3 s period, averaged across each group (floor effect). For purposes of clarity, mean values are displayed without error bars, and the relaxation condition for the non-meditators is labeled as meditation.

The pattern of findings reported above also held true for the highest and lowest heart rates observed during epochs 1, 2, and 3. Specifically, increases in the highest (and lowest) heart rates occurred for conditions containing increasing doses of isoproterenol (but not saline), in the absence of any group differences whatsoever. In the interest of brevity the relevant statistics have not been reported here, but rather, the reader is referred to Figure 3.

### Automated heart rate and blood pressure

#### Automated heart rate

We observed a significant effect of condition on the automated measure of heart rate *F*_(1, 3)_ = 115.88, *p* < 0.0001, η^2^_*p*_ = 0.84, ε = 0.813, observed power = 1.00, as well as dose *F*_(1, 4)_ = 74.42, *p* < 0.0001, η^2^_*p*_ = 0.77, ε = 0.909, observed power = 1.00. There was also a significant interaction between condition and dose *F*_(1, 12)_ = 40.31, *p* < 0.0001, η ^2^_*p*_ = 0.65, ε = 0.438, observed power = 1.00, suggesting that increases in automated heart rate occurred at increasing doses of isoproterenol (but not saline) administration. There were again no group differences in heart rate during this period. There was no effect of group *F*_(1, 28)_ = 0.06, *p* = 0.80, η ^2^_*p*_ = 0.00, observed power = 0.06, and there were no interactions between condition and group *F*_(1, 3)_ = 1.02, *p* = 0.38, η ^2^_*p*_ = 0.04, observed power = 0.27, between dose and group *F*_(1, 4)_ = 2.12, *p* = 0.09, η ^2^_*p*_ = 0.09, observed power = 0.61, or between condition and group and dose *F*_(1, 12)_ = 1.19, *p* = 0.32, η ^2^_*p*_ = 0.05, observed power = 0.68, suggesting that the mean heart rate increases observed for increasing doses of isoproterenol with the automated measure were similar between the groups (Figure 2C).

#### Systolic blood pressure

**Figure 4:**
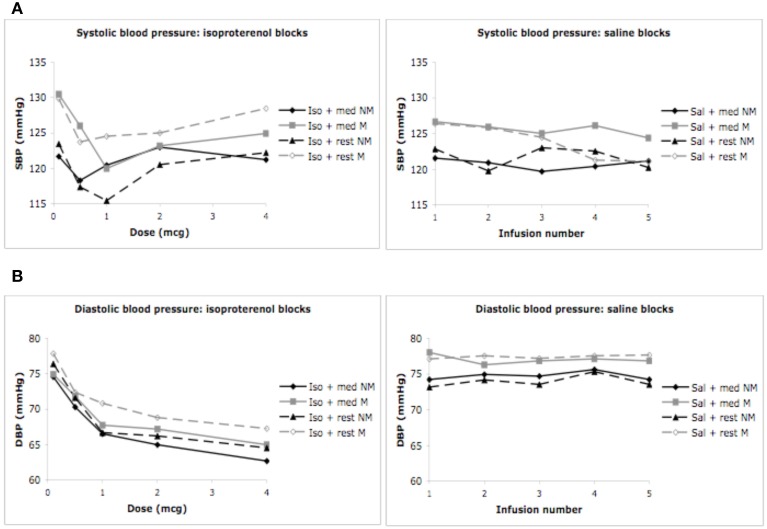
**Blood pressure for both groups during meditation/relaxation and rest. (A)** Systolic blood pressure measured via automated blood pressure monitor. **(B)** Diastolic blood pressure measured via automated blood pressure monitor. For purposes of clarity, mean values are displayed without error bars, and the relaxation condition for the non-meditators is labeled as meditation.

We did not observe a significant effect of condition *F*_(1, 3)_ = 0.03, *p* = 0.99, η^2^_*p*_ = 0.00, observed power = 0.06, but did observe a significant effect of dose *F*_(1, 4)_ = 4.35, *p* = 0.006, η^2^_*p*_ = 0.17, ε = 0.820, observed power = 0.82, on the automated measure of systolic blood pressure. There was no interaction between condition and dose *F*_(1, 12)_ = 1.74, *p* < 0.08, η ^2^_*p*_ = 0.07, observed power = 0.87, suggesting that changes in systolic blood pressure were not influenced by isoproterenol administration. No group differences in systolic blood pressure were observed. There was no effect of group *F*_(1,28)_ = 0.71, *p* = 0.41, η^2^_*p*_ = 0.03, observed power = 0.13, and there were no interactions between condition and group *F*_(1, 3)_ = 0.80, *p* = 0.50, η ^2^_*p*_ = 0.04, observed power = 0.21, between dose and group *F*_(1, 4)_ = 1.52, *p* = 0.21, η ^2^_*p*_ = 0.07, observed power = 0.45, or between condition and group and dose *F*_(1, 12)_ = 1.03, *p* = 0.42, η ^2^_*p*_ = 0.05, observed power = 0.59. These decreases in systolic blood pressure were similar for both groups and appeared to occur during the first few doses, irrespective of condition, suggesting that these effects were likely not related to isoproterenol (Figure 4A).

#### Diastolic blood pressure

We observed a significant effect of condition *F*_(1, 3)_ = 25.37, *p* < 0.0001, η ^2^_*p*_ = 0.54, observed power = 1.00, and dose *F*_(1, 4)_ = 28.58, *p* < 0.0001, η^2^_*p*_ = 0.57, ε = 0.653, observed power = 1.00 on the automated measure of diastolic blood pressure. There was also a significant interaction between condition and dose *F*_(1, 12)_ = 12.43, *p* < 0.0001, η^2^_*p*_ = 0.36, ε = 0.692, observed power = 1.00, suggesting that changes in diastolic blood pressure occurred at increasing doses of isoproterenol (but not saline) administration. There were again no group differences in diastolic blood pressure. There was no effect of group *F*_(1, 28)_ = 0.65, *p* = 0.43, η ^2^_*p*_ = 0.03, observed power = 0.12, and there were no interactions between condition and group *F*_(1, 3)_ = 0.29, *p* = 0.82, η ^2^_*p*_ = 0.01, observed power = 0.10, between dose and group *F*_(1, 4)_ = 0.36, *p* = 0.76, η ^2^_*p*_ = 0.02, observed power = 0.13, or between condition and group and dose *F*_(1, 12)_ = 0.45, *p* = 0.90, η ^2^_*p*_ = 0.02, observed power = 0.25. These findings suggest decreases in diastolic blood pressure were similar for both groups at increasing doses of isoproterenol (Figure 4B). Such a decrease in diastolic blood pressure is consistent with the vasodilatory effects of isoproterenol.

### Individual heart rates

**Figure 5:**
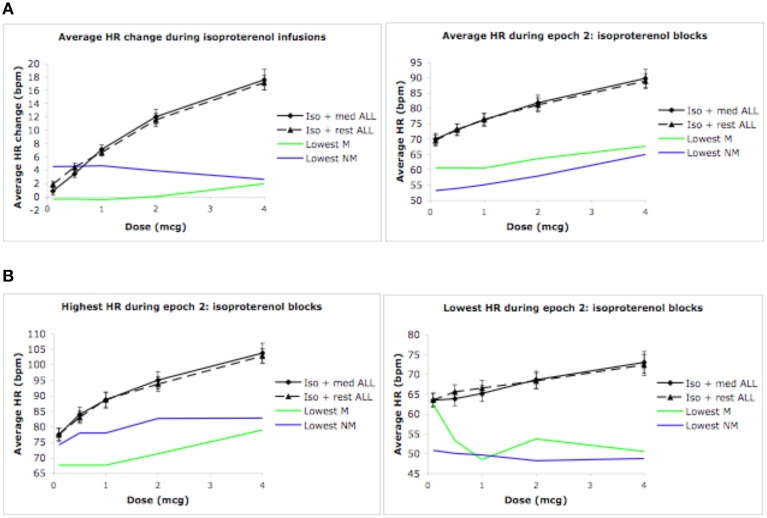
**Global and individual outlier heart rate changes during isoproterenol conditions. (A)** Global mean heart rate change (epoch 2 minus epoch 1), and global mean heart rate observed during epoch 2 for all participants. **(B)** Global maximum heart rate change and global minimum heart rate change observed during epoch 2 for all participants. The green lines indicate the meditator who displayed the lowest response during the isoproterenol plus meditation condition. The blue lines indicate the non-meditators who displayed the lowest response during the isoproterenol plus relaxation condition. For purposes of clarity, the relaxation condition for the non-meditators is labeled as meditation. Error bars represent standard error of the mean.

Given the absence of group effects, an examination of individual heart rate changes during the isoproterenol plus meditation condition was performed. The goal was to identify whether any reductions in heart rate similar those reported in Dimsdale and Mills (2002) had occurred in individual meditators or non-meditators. At the individual level, we examined mean heart rate changes during epoch 2, mean heart rate during epoch 2, and lowest and highest heart rate during epoch 2 derived from the continuous heart rate waveform during the isoproterenol plus meditation/relaxation and isoproterenol plus rest conditions. Based on the hypothesis that meditation would result in a reduced response to isoproterenol, individual meditators and non-meditators displaying the lowest responses were selected for comparison with their respective groups. As Figure 5A indicates, there were individuals in each group who displayed a reduced heart rate response and reduced average heart rates relative to their group averages. Similarly, there were individuals in each group who displayed reduced heart rate maxima and minima when compared with their group averages (Figure 5B). However, although these reflect large differences in the magnitude of the response to isoproterenol (compared to the respective group mean), only one individual (a meditator) appeared to display a reduced heart rate at increasing doses, and only when using the criterion for lowest heart rate observed during a 3 s period.

### Subjective experience

Similarity ratings of the meditation and relaxation practices did not differ between the groups *F*_(1, 25)_ = 0.03, *p* = 0.86, η ^2^_*p*_ = 0.001, observed power = 0.054^2^. The meditation and relaxation practices appeared to be rated as more similar to the usual practice during the saline infusion condition, but this difference was not statistically significant *F*_(1, 1)_ = 2.18, *p* = 0.15, η ^2^_*p*_ = 0.08, observed power = 0.30.

### Post analysis

Since a heterogeneously recruited sample of meditators could potentially result in differential heart rate responses during meditation, we subsequently evaluated the responses of the Kundalini and Vipassana practitioners separately, and in comparison to each other.

### Vipassana vs. healthy comparison

During the meditation condition there was no effect of group *F*_(1, 24)_ = 1.31, *p* = 0.26, η ^2^_*p*_ = 0.05, observed power = 0.20, and there were no interactions between condition and group *F*_(1, 1)_ = 0.002, *p* = 0.98, η ^2^_*p*_ = 0.00, observed power = 0.05, or between condition and group and dose *F*_(1, 4)_ = 0.10, *p* = 0.98, η ^2^_*p*_ = 0.004, observed power = 0.07. This suggests that during both meditation conditions (saline and isoproterenol) the Vipassana meditators displayed similar average heart rate increases as healthy comparisons.

### Kundalini vs. healthy comparison

During the meditation condition there was no effect of group *F*_(1, 17)_ = 2.1, *p* = 0.17, η ^2^_*p*_ = 0.11, observed power = 0.27, and there were no interactions between condition and group *F*_(1, 1)_ = 0.006, *p* = 0.94, η ^2^_*p*_ = 0.00, observed power = 0.05, or between condition and group and dose *F*_(1, 4)_ = 1.58, *p* = 0.19, η ^2^_*p*_ = 0.09, observed power = 0.46. This suggests that during both meditation conditions (saline and isoproterenol) the Kundalini meditators displayed similar average heart rate increases as healthy comparisons.

### Kundalini vs. vipassana

During the meditation condition there was no effect of group *F*_(1, 13)_ = 0.11, *p* = 0.75, η ^2^_*p*_ = 0.008, observed power = 0.06, and there were no interactions between condition and group *F*_(1, 1)_ = 0.001, *p* = 0.98, η ^2^_*p*_ = 0.00, observed power = 0.05, or between condition and group and dose *F*_(1, 4)_ = 0.88, *p* = 0.48, η ^2^_*p*_ = 0.06, observed power = 0.26. This suggests that during both meditation conditions (saline and isoproterenol) the two groups of meditators displayed similar average heart rate increases.

## Discussion

As expected, bolus isoproterenol infusions produced dose-dependent increases in heart rate in both groups during a condition of rest. However, meditators did not display lowered heart rate responses to isoproterenol while practicing meditation. Both meditators and non-meditators displayed similar dose dependent increases in heart rate during conditions of isoproterenol plus rest, and during isoproterenol plus meditation/relaxation. The lack of group differences in heart rate was reliable. It was observed for five different measures of heart rate (mean heart rate change, mean heart rate, lowest heart rate during a 3 s period, highest heart rate during a 3 s period, and via automated heart rate monitor), and throughout three epochs that captured the entire time span over which isoproterenol induced changes occur. Equivalent decreases in diastolic blood pressure were also observed in both groups following increasing doses of isoproterenol. These decreases are consistent with the known vasodilatory effects of isoproterenol on lowering diastolic blood pressure. We do not consider these to be related to the meditation intervention, as they occurred in both isoproterenol conditions. Non-meditators did not display lowered heart rates when practicing a relaxation condition, which was predicted.

Since the Dimsdale and Mills (2002) study reported a finding in a single individual, we examined individual heart rate responses within in each group of participants. Of the measures utilized, there was only one participant who demonstrated an appreciable reduction in heart rate during the meditation plus isoproterenol condition similar to that reported by Dimsdale and Mills (2002) (i.e., a 17 bpm decrease in heart rate from the lowest to the highest dose). This finding occurred in a meditator, but it was only observed with one measure, the lowest heart rate, which is also the least reliable index of the response to isoproterenol. This finding was no longer present when more robust measures of the heart rate response to isoproterenol, such as the average heart rate and average heart rate change, were examined in the same individual. Thus, it seems unlikely that the individual effect reported by Dimsdale and Mills (2002), although intriguing, generally applies to individuals practicing meditation.

Several potential factors might explain the discrepancy in findings between the two studies. First, individual variability in heart rate responses might explain their observation of lowered heart rate in one subject. We observed considerable variability in heart rate responses to isoproterenol even when using a continuous measure, with select individuals from both groups exhibiting attenuated heart rate responses to isoproterenol. A second and related reason could be measurement error. Dimsdale and Mills (2002) did not utilize a continuous measure of heart rate in their original study, and it is unclear when the automated monitor they utilized actually captured their meditator's heart rate. Although they report that heart rate was measured 60 s after the infusion was delivered, it is possible that there was enough measurement variability to result in missing the heart rate epoch of interest. In the present study, we observed large variability in the output of the automated heart rate measurement when using the blood pressure monitoring device (e.g., 58–93 s after initiating measurement), which is long enough to potentially miss capturing an individual's heart rate response. It is also possible that the window of measurement of the automated heart rate monitor was too brief. In the current study, we observed the largest reductions in heart rate response during a momentary measure, the lowest heart rate during a 3 s period, which provides some plausibility to this theory. Third, their meditator was not formally trained, and indicated that she would often enter such deep states that she would need to set an alarm clock to rouse her from meditation. Thus, it is possible that this individual likened sleep (or some other altered state of consciousness) with meditation, and that the observed effect had nothing to do with meditation but a different process worth understanding further. However, despite these potential methodological and conceptual considerations, the participant in Dimsdale and Mills' study did demonstrate a heart rate increase at the 1.0 mcg dose, followed by two successive decreases below her resting heart rate. Assuming this was not a spurious finding, such a profound decrease in heart rate could also occur during an episode of increased vagal output (as suggested by the authors). Neurocardiogenic syncope is one example of such an increase in vagal output. These episodes are usually preceded by an increase in sympathetic tone (as was observed in the meditator at the 1.0 mcg dose) and can even be triggered by isoproterenol (Kikushima et al., 1999). These episodes are often foreshadowed by symptoms such as lightheadedness, nausea, warmth, pallor and/or sweating, and although the meditator denied experiencing a subset of these (nausea, dizziness or fainting), it is possible that she was not aware of these symptoms given the fact that she was in a meditative state described as so deep as to require rousing with an alarm clock. Since heart rate was the only autonomic measure reported in that study, it is difficult to determine whether such a reflex occurred. Evaluating this particular meditator's (or others the future) cardiovascular responses to upright tilt table testing (perhaps even incorporating isoproterenol) could be useful to exclude this as a possibility. Another possibility is that respiratory modulation could have played a role in the observed heart rate changes, as respiratory induced changes in heart rate variability have been observed in meditators (Peng et al., 2004). We did not examine this possibility as it was outside the scope of inquiry in our study.

There are several limitations associated with the present study. First, the meditation practice was investigated under highly artificial circumstances. Meditators were meditating in a novel environment, with an intravenous line placed, were attached to various recording devices and were seated next to two individuals who were observing them throughout the study. Despite efforts to facilitate the practice, such as a curtain between the investigators and participant, and a dimly lit, quiet room, it cannot be claimed that the meditation and relaxation conditions took place in the usual environment. Second, the current study does not take into account the potential influence of testing anxiety, that is, concern on the part of the meditators about “performing well.” Although meditators did not voice any such concerns, and did not differ from the non-meditators in questionnaire measures of anxiety, this possibility cannot be ruled out. A third limitation is the fact that the meditation plus saline conditions did not appear to lower the heart rate substantially. This could again be due to the artificiality of the environment, thus preventing meditators from authentically engaging in their practice and displaying increased parasympathetic indices. However, retrospective ratings of the quality of the meditation practice indicated that meditators found their meditation practice during the infusions to be predominantly similar to their daily practice, reducing this as a possibility. The lack of heart rate reductions during saline could also be related to the type of meditation participants practiced. For example, some meditations are suggested to increase cardiovascular states whereas others are suggested to decrease them (Amihai and Kozhevnikov, 2014). Since most meditators were not instructed to use their meditation practice to reduce their heart rate (this would have been seen by the Vipassana practitioners as constraining and interfering with the meditation practice), it is possible that the mediators were performing the former type of meditation. Reports from the meditators about the nature and quality of their practice did not seem to indicate this as a possibility.

It is also possible that the lack of attenuated autonomic responses in the current study could be related to a non-ideal sample of meditators and/or a non-ideal meditation practice. For example, since the majority of the meditators came from a Vipassana background, their default meditation practice may not be intended to have effects on autonomic state. This argument is somewhat limited by the fact that Vipassana meditation techniques emphasize the cultivation of quiescent and tranquil states of mind, and show some evidence of altered sympatho-vagal balance (Krygier et al., 2013). Alternatively, it is theoretically possible that the default body scanning meditation practice practiced by the Vipassana meditators in this study could have been similar to a form of “open presence” rather than “focused attention” meditation. Since the former type of meditation has been associated with heightened arousal instead of relaxation, this might be expected to induce an increased autonomic response during meditation instead of a quiescent one. We observed a few marginal interactions between condition and group on heart rate with the meditators potentially exhibiting increased heart rates during meditation, that might potentially support this possibility. However, these non-significant effects were not consistently observed across the numerous heart rate measures employed. Further complicating this theoretical interpretation is evidence suggesting that open presence meditation can be associated with attenuated autonomic responses to startle challenges that probe sympathetic reactivity (Levenson et al., 2012). We conclude there is no direct evidence in the current study to support the notion of differentially decreased or increased autonomic indices in the meditators.

Another question about the current sample is whether the absence of effect could have been due to an inexperienced sample of meditators relative to the meditator in Dimsdale and Mills (2002), as the minimum requirement was only 2 years of meditative experience. Direct comparison between the studies is not possible, as the years of practice were not provided in that report. However, the notion that duration of practice is synonymous with meditative expertise is a matter of ongoing debate. Previous investigations of long term meditators have not always yielded evidence of increased ability, even for aspects of internal sensory experience that are routinely cultivated in Vipassana and Kundalini traditions (Khalsa et al., 2008; Daubenmier et al., 2013). A larger issue is the fact that the meditator described in that report was not formally trained in any particular tradition, making it impossible to select an appropriately representative group of meditators. In the current study we recruited formally trained meditators from multiple traditions, used extensive measurements of heart rate response, and examined individual outlier responses in both groups to determine whether there was specific evidence that meditation was associated with a lowered heart rate. Though some individuals displayed attenuated heart rate responses to isoproterenol, we found no evidence differentiating meditators from non-meditators.

A general point is that while these limitations warrant consideration, many of them would be similarly imposed by any empirical study of meditation and thus cannot be easily obviated. If further investigations of the current topic were continued, one helpful strategy would be to screen large samples of meditators to identify those individuals who can reliably demonstrate enhanced autonomic regulatory capabilities, and then perform detailed investigation into the cognitive and neurophysiological mechanisms underlying such abilities.

We feel it is also important to note that the current findings do not necessarily negate previously reported findings of decreased autonomic tone following the practice of meditation. Although our study appeared sufficiently powered to detect effects related to the isoproterenol vs. saline conditions, and the impact of different doses, there was lower power at the group level. While replicating this approach with a larger sample would help to address this limitation, the current results suggest that any effects, even if found reliable at the population level, would be small and would have limited consequence at the individual level. The current findings also have limited bearing on the effects of meditation on emotional experience or emotion regulation, effects consistently perceived at the individual level with training, as these constructs were not investigated. High levels of adrenergic hormones are often associated with acute anxiety, stress, anger, fear and hostility, and different forms of meditation are routinely practiced by some individuals to help ameliorate such experiences (Shirey, 2007; Robins et al., 2012; Serpa et al., 2014). Determining the biological mechanisms supporting those beneficial effects remains an important and active area of inquiry, regardless of whether they are derived from changes in autonomic reactivity or from changes in the central nervous system. Overall, these results simply suggest that the formal act of meditation may not be sufficient to physiologically counteract certain forms of elevated levels of peripheral cardiovascular adrenergic arousal or engender quicker recovery from such arousal.

## Support

This research was supported by a grant from the National Center for Complementary and Alternative Medicine (NCCAM F31 AT003061-01A1), the National Center for Research Resources, General Clinical Research Centers Program (NIH M01-RR-59), a McDonnell Foundation Collaborative Award to DT (#220020387), and the William K. Warren Foundation.

### Conflict of interest statement

The authors declare that the research was conducted in the absence of any commercial or financial relationships that could be construed as a potential conflict of interest.

## Footnotes

^1^These measures were incorporated after several participants had been tested. As a result, data for the full sample are unavailable (measures were collected from 11 meditators and 13 non-meditators).

^2^Data were missing from 3 healthy comparison participants.
